# Does the Maxim “Once a Caesarean, Always a Caesarean” Still Hold True?

**DOI:** 10.1371/journal.pmed.0020305

**Published:** 2005-09-27

**Authors:** Austin Ugwumadu

## Abstract

Ugwumadu discusses a new tool, reported in *PLoS Medicine,* that can help to predict the risks of Caesarean section and uterine rupture in women attempting vaginal birth after prior Caesarean section.

## Teaser

A new tool can help to predict the risks of Caesarean section and uterine rupture in women attempting vaginal birth after prior Caesarean section

Edwin Cragin's century-old opinion, “once a Caesarean, always a Caesarean,” is correct if placed in proper context [1]. Cragin was predicting the near certainty of repeat Caesarean section in a self-selected group of women (1%–2%) who failed to deliver vaginally after several days in active labour. At that time, rickets and pelvic deformity were prevalent even in industrialised countries, syntocinon for augmentation of slow labour was unknown, and surgery was crude and dangerous. The primary Caesarean section was undertaken to save the life of an exhausted, dehydrated, ketotic, often pyrexial and delirious, moribund mother. In those days, fetal compromise was not an indication for Caesarean section; indeed there was no such thing as fetal monitoring (either antepartum or intrapartum). Cragin recognised that women who survived one Caesarean section were not candidates for vaginal delivery in subsequent pregnancies.

## The Rising Rate of Caesarean Sections

Within the last three decades, Caesarean section rates in many countries have risen 5-fold to 10-fold. For example, in England and Wales the rate rose from 4% in 1970 to a current 22% [2]. In the United States it rose from 6% in 1970 to 24% in 1990 [3], while in the municipality of Ribeirao Preto, State of Sao Paulo, Brazil, the rate rose from 30.3% in 1978–1979 to 50.8% in 1994 [4]. The rising rate has been driven at least in part by our reliance on electronic fetal monitoring, pressure from health consumers to salvage small babies even at the very margins of viability, fear of litigation, decreasing expertise in operative vaginal deliveries and, in the West, lifestyle choices.

The current Caesarean section rate of about 50% in some countries [4] is too high and unsustainable, and according to the World Health Organization, is not associated with any further improvement in perinatal outcome compared to outcomes at a Caesarean section rate of 10%–15% [5]. Can we halt and reverse this trend, reduce the morbidity and drain on health care budgets associated with it, and, above all, balance maternal choice issues? In contrast to Edwin Cragin's patients, women today are healthier (rickets is rare now, even in nonindustrialised countries), and in the rich world at least, oxytocin, blood transfusion, antibiotics, and thromboprophylaxis are available, while surgery and anaesthesia are safe. Therefore, some obstetricians have enthusiastically and nonselectively promoted vaginal birth after Caesarean section. However, the consequences in inappropriate cases can be disastrous.

## The Risks of Labour after Caesarean Section

Labour/vaginal birth after Caesarean section is associated with increased risks of uterine rupture and feto-maternal morbidity and mortality. These risks and costs of care rise further if the attempt fails [6]. Available evidence suggests that the complication rates are lowest in women whose attempt at vaginal delivery after Caesarean has been successful—even lower than in women who had planned Caesarean section. Therefore, the crucial questions are how to reliably predict successful attempt at vaginal birth after Caesarean section, and how to determine and quantify the magnitude of the risk of failure that is acceptable to women and their caregivers.

In this issue of *PLoS Medicine*, Smith and colleagues aimed to develop a prediction tool to predict the likelihood of Caesarean section and uterine rupture in women with one prior Caesarean undergoing a trial of labour [7]. Such a tool would be useful for counseling women and for policy makers and health care commissioners. The authors randomly allocated 23,286 women (from the linked Scottish Morbidity Record [SMR2] and the Scottish Stillbirth and Neonatal Death Enquiry) to two groups: a model development group and a model validation group. None of the previously published prediction tools had been validated prospectively. The authors' model ranked women as high risk of emergency Caesarean section (≥ 40%), or low risk (≤ 20%), and the primary analysis was confined to women who delivered ≥ 40 weeks' gestation.

The factors that were associated with Caesarean section, based on the multivariate analysis, are shown in Table 1. The predicted risk of Caesarean section was also associated with the risk of uterine rupture (odds ratio [OR] 1.22, for a 5% increase in predicted risk, 95% confidence interval [CI] 1.14–1.31) and rupture associated with perinatal death (OR 1.32 for a 5% increase in predicted risk, 95% CI 1.02–1.73). In the validation group, 10.9% of the women predicted to have a low risk of having Caesarean section actually had a Caesarean section, while 47.7% of women predicted to have a high risk of Caesarean section had one. The incidence of uterine rupture was 2.0 and 9.1 per 1000 in the low and high-risk categories respectively (OR 4.5; 95% CI 2.6–8.1).

**Table 1:**
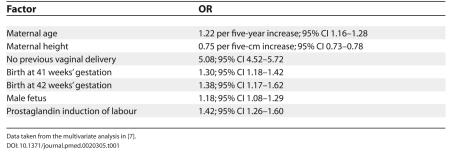
Factors Associated with Caesarean Section in Women with One Prior Caesarean Undergoing a Trial of Labour

Data taken from the multivariate analysis in [7].

## The Value of the New Predictive Tool

When applied to the total population, the predictive model classified just over half (52.5%) of the study population into a low (36%) and a high (16.5%) likelihood of Caesarean. Over half of those predicted to be at high risk of Caesarean section did not have one, suggesting that the value of this tool may well lie in predicting those patients who are unlikely to have an emergency Caesarean and/or uterine rupture (with or without perinatal death).

At best, the tool classifies about half our obstetric population.

By confining the primary analysis to women who delivered at ≥ 40 weeks' gestation, the authors aimed to capture only the women who “truly” intended to have a vaginal delivery, assuming that planned Caesarean deliveries would have been undertaken by 39 weeks' gestation. There is some evidence, however, that the likelihood of successful vaginal delivery decreases beyond 40 weeks' gestation in women with prior Caesarean section [8,9], raising the question of how generalisable these findings are to a significant proportion of women with previous Caesarean section who go into spontaneous labour between 37 and 40 weeks' gestation. Not surprisingly, when the model was applied to this latter group in a secondary analysis, the risk of Caesarean section was found to be 18%, a rate predictably lower than the chosen cutoff for low risk of Caesarean section. It would seem reasonable, therefore, to discuss this low risk with women and indeed to encourage them to proceed with a trial of vaginal delivery if spontaneous labour supervenes before 40 weeks.

Even in the high-risk group, the risk of uterine rupture was less than 1% (9.1 per 1000). One question is whether this magnitude of risk justifies a repeat Caesarean section in modern units that deploy round-the-clock obstetric, pathology, and anaesthetic services, practise continuous electronic fetal monitoring, and have facilities for emergency Caesarean section.

## The Issue of Women's Choice

The more fundamental 21st century question is whether a previous Caesarean section is a medical indication for a repeat Caesarean. If the answer is “yes,” then women can truly exercise the choice to have or not to have one. If the answer is “no,” then true choice hardly exists, at least not in the public sector. The right to choose a Caesarean may then adversely affect the rights and just expectations of other women and their babies who medically need operative delivery. Autonomous clinicians, conscious of responsible utilisation of health care resources, will decline what is arguably then a lifestyle choice. Guidelines from The National Institute for Clinical Excellence (http://www.nice.org.uk) on Caesarean section recommend that “maternal request is not on its own an indication for Caesarean section,” and that “a clinician can decline such a request” [10]. The guidelines, however, remain silent on how this applies to women with previous Caesarean section. For example, are obstetricians liable if they persuade a woman to have a trial of labour, and complications occur?

Smith et al. should be applauded for developing their new tool for the prediction of Caesarean section and uterine rupture in women with previous Caesarean. It is a huge step forward, but it is not the definitive tool. At best, it classifies just about half our obstetric population. The critical questions of women's choice and the medical view of labour after Caesarean section in the 21st century remain unanswered.

## Abbreviations

CI: confidence interval
OR: odds ratio
